# Expression characteristics and prognostic value of mycoplasma pneumoniae DNA, Interleukin-6, and Interleukin-10 levels in children with mycoplasma pneumoniae pneumonia

**DOI:** 10.12669/pjms.42.7.14948

**Published:** 2026-07

**Authors:** Yan Cheng, Juanjuan Wang, Zhiyong Chen

**Affiliations:** 1 Yan Cheng, Department of Pediatrics, The Affiliated Hospital 6 of Nantong University, Department of Pediatrics, Yancheng Third People’s Hospital, Yancheng, Jiangsu Province 224000, P.R. China; 2 Juanjuan Wang, Department of Pediatrics, The Affiliated Hospital 6 of Nantong University, Department of Pediatrics, Yancheng Third People’s Hospital, Yancheng, Jiangsu Province 224000, P.R. China; 3 Zhiyong Chen, Department of Pediatrics, The Affiliated Hospital 6 of Nantong University, Department of Pediatrics, Yancheng Third People’s Hospital, Yancheng, Jiangsu Province 224000, P.R. China

**Keywords:** Expression characteristics, IL-6, IL-10, *Mycoplasma* pneumoniae pneumonia, MP-DNA, Prognostic value

## Abstract

**Objectives:**

To investigate the expression characteristics and prognostic value of *Mycoplasma pneumoniae* DNA (MP-DNA), interleukin-6 (IL-6), and interleukin-10 (IL-10) levels in children with *Mycoplasma* pneumoniae pneumonia (MPP).

**Methodology:**

This was a retrospective analysis of two hundred children who were suspected of having MPP from January 2022 to December 2024 at Yancheng Third People’s Hospital. On the basis of the clinical diagnosis and results of the *Mycoplasma pneumoniae* culture, the patients were divided into a study group (with MPP, n=103) and a control group (without MPP, n= 97). The study group was divided into a good prognosis group (n=82) and a poor prognosis group (n=21) on the basis of whether they experienced complete resolution of symptoms after treatment, severe complications, or recurrence during the 3-month follow-up. Serum levels of MP-DNA, IL-10, and IL-6 were determined, and their levels in patients with good/poor prognosis were statistically analyzed to explore the risk factors for poor prognosis in patients with MPP.

**Results:**

Compared with those in the control group, levels of MP-DNA (t=29.033; p<0.0001), IL-10 (t=18.233; p<0.0001), and IL-6 (t=28.692; p<0.0001) in the study group were greater (P<0.05). MP-DNA (t=27.174; p<0.0001), IL-10 (t=8.400; p<0.0001), and IL-6 (t=8.981; p<0.0001) levels were higher in the poor prognosis group than in the good prognosis group (P<0.05). High levels of MP-DNA, IL-10, and IL-6 are important risk factors for poor prognosis in children with MPP (P<0.05). Compared with individual diagnosis, combined detection of MP-DNA, IL-10, and IL-6 is more effective.

**Conclusion:**

Children with MPP have elevated levels of MP-DNA, IL-10, and IL-6, and their high expression is a risk factor for poor prognosis.

## INTRODUCTION

*Mycoplasma pneumoniae* pneumonia (MPP), a common cause of community-acquired pneumonia in children, is characterized by rapid progression and a high incidence of complications.^1^,^2^ In clinical practice, the prognosis of children with MPP is mostly judged on the basis of traditional indicators such as symptoms and imaging findings. However, these indicators are often insufficiently specific and have limited early predictive value, which may easily lead to delayed treatment or overtreatment.^3^–^5^

*Mycoplasma pneumoniae* DNA (MP-DNA) is a molecular marker that directly reflects pathogen infection and is correlated with the severity of infection.^6^ Among children with a higher load of MP-DNA, a stronger pulmonary inflammatory immune response was identified.^7^ In addition, the MP-DNA level achieved a specificity of 92.19% and a sensitivity of 97.62% in distinguishing MPP and normal children, and the area under the curve was 0.979. This study indicated that MP-DNA is a promising indicator of the development and progression of MPP.^8^ Interleukin-6 (IL-6) and interleukin-10 (IL-10) are key cytokines involved in the inflammatory response. As a proinflammatory cytokine, IL-6 participates in the pathological process of pulmonary injury, such as pancreatitis-induced acute lung injury and ventilator-induced lung injury.^9^-^10^ IL-10 often exerts anti-inflammatory and immunomodulatory effects.^11^ Studies have shown that the imbalance between these two cytokines plays an important role in the progression of MPP.^12^–^14^ For example, Wang et al. reported that serum IL-6 was associated with lobar lesions in pediatric MPP patients, which is a pathological change prior to lung necrosis.^12^

Although current studies have revealed significant alterations in the expression of MP-DNA, IL-6, and IL-10 in children with MPP, their ability to predict the prognosis of pediatric MPP needs to be investigated in clinical cases. Here, we speculated that the MP-DNA, IL-6, and IL-10 contents were outcome indicators of children with MPP and that their combination can achieve better predictive efficiency. A retrospective study was designed to assess the expression levels of MP-DNA, IL-6, and IL-10 in the peripheral blood of children with MPP to explore the predictive value of their combined application. These results may provide a scientific basis for early risk stratification, optimization of treatment regimens, and prognostic evaluation of MPP.

## METHODOLOGY

The clinical data of two hundred children with suspected MPP admitted to Yancheng Third People’s Hospital from January 2022 to December 2024 were retrospectively analyzed. On the basis of the combination of comprehensive clinical diagnosis and results of *Mycoplasma pneumoniae* culture (the gold standard of MPP diagnosis) for grouping, children diagnosed with MPP were assigned to the study group (n=103), and non-MPP children were assigned to the control group (n=97).

### Inclusion criteria:

- Aged 1–14 years, with clinical manifestations of respiratory symptoms such as fever and cough.
- Pulmonary imaging examinations (chest X-ray or CT) suggest pneumonia, which is consistent with the clinical characteristics of suspected MPP.
- Completed *Mycoplasma pneumoniae* culture, peripheral blood MP-DNA detection, and IL-6 and IL-10 detection after admission.
- In the study group, positive *Mycoplasma pneumoniae* culture and etiological examinations (viral nucleic acid/antigen, bacterial culture, etc.) excluded infections by other pathogens.
- In the control group, a negative *Mycoplasma pneumoniae* culture was performed, and etiological examinations confirmed a single nonmycoplasma pathogen infection (such as a virus, bacteria, or chlamydia), or a comprehensive clinical diagnosis confirmed noninfectious lung diseases (such as aspiration pneumonia and hypersensitivity pneumonia).
- Complete clinical data, including demographic information, symptoms and signs, laboratory examinations, imaging data, treatment plans, and follow-up records, were obtained.

### Exclusion criteria:

- Children with mixed infections of *Mycoplasma pneumoniae* and other pathogens (bacteria, viruses, fungi, etc.).
- Children with underlying diseases such as congenital cardiopulmonary diseases, immune deficiency, liver and kidney dysfunction, and hematological diseases.
- Children who have received medications such as macrolide antibiotics, glucocorticoids, and immunosuppressants may have different detection indicators or disease diagnoses during the course of the disease.
- Children with severe complications (such as sepsis and multiple organ failure) who could not complete standardized treatment and follow-up were excluded.

### Ethical approval:

The study was approved by the ethics committee of Yancheng Third People’s Hospital (#2022–340; dated November 1, 2022). Owing to the retrospective nature of the study, informed consent was waived.

### Observation indicators:

The following information was collected from all patients: gender, age, duration of fever, pulmonary imaging findings (presence of pulmonary consolidation/pleural effusion), number of bronchoscopy treatments, and length of hospital stay. All patients underwent MP-DNA, IL-10, and IL-6 detection in peripheral blood samples as follows:

### MP-DNA detection:

Nucleic acids were extracted from 5 ml of peripheral blood, and the MP DNA load was assessed by real-time quantitative polymerase chain reaction (qRT-PCR). An *M. pneumoniae* DNA fluorescence diagnostic kit (Sansure Biotech, Shanghai, China) was used. A real-time fluorescent quantitative PCR instrument (SLAN-96P, Hongshi, Shanghai, China) was used to measure DNA content. The operations strictly followed the kit instructions. Briefly, 100 μL of anticoagulated whole blood was added to nucleic acid extraction reagents, and genomic DNA was obtained after lysis and purification. The reaction conditions were as follows: predenaturation at 95°C for five minutes, followed by 40 cycles of denaturation at 95°C for 15 seconds and annealing/extension at 60°C for 30 seconds, and a final extension at 72°C for 5 minutes. The specific copy number of each sample was recorded.

### IL-6 and IL-10 detection:

The enzyme-linked immunosorbent assay (ELISA) method was used to measure IL-6 and IL-10 levels in frozen serum samples from 3 ml of peripheral blood. A microplate reader, a human IL-6 ELISA detection kit (#CSB-E04638h; CUSABIO, Wuhan, China), and a human IL-10 ELISA detection kit (#CSB-E04593h; CUSABIO, Wuhan, China) were used. Frozen serum samples were thawed at room temperature and mixed well before testing. ELISA was performed in accordance with the instructions of the kit: standards, samples, and corresponding antibodies were added separately, the samples were incubated at 37°C for 60 minutes, and the samples were washed five times with washing buffer; then, the enzyme-labeled conjugates were added, and the samples were incubated at 37°C for 30 minutes. After rewashing, chromogenic substrate was added, and the samples were incubated at 37°C in the dark for 15 minutes. Finally, a stop solution was added to terminate the reaction. The absorbance (OD value) of each well was measured at 450 nm using a microplate reader. A standard curve was plotted based on the standard concentrations and their corresponding OD values to calculate the concentrations of IL-6 and IL-10.

The levels of MP-DNA, IL-10, and IL-6 were compared (1) between the study group and the control group and patients in the study group stratified by prognosis. Good prognosis: Complete resolution of symptoms after treatment, no complications, and no recurrence during the 3-month follow-up; poor prognosis: Treatment failure requiring regimen adjustment, occurrence of complications, or recurrence during follow-up. Receiver operating characteristic (ROC) curves were generated to evaluate the predictive value of single and combined detection of MP-DNA, IL-6, and IL-10 for poor prognosis of MPP, and the area under the curve (AUC), sensitivity, and specificity of each indicator were calculated.

### Statistical analysis:

Data were processed using SPSS 22.0 software. Categorical data are described as counts and were analyzed by the χ² test. Continuous data were tested for homogeneity of variance (Bartlett test) and normality (Kolmogorov-Smirnov test), and since they met the assumptions of homogeneity of variance and approximate normality, they were described as the mean ± standard deviation () and analyzed using the t test. Logistic regression analysis was used to identify the risk factors for MPP prognosis, and a predictive model was established using R 3.6.1 (Windows version). The significance level was set at α = 0.05.

## RESULTS

This study retrospectively analyzed the data of two hundred pediatric patients with suspected MPP. The study group included 103 patients, 56 males and 47 females, aged 3–12 years, with a mean age of 6.40±1.93 years. In the control group, there were 52 males and 45 females, aged 2–13 years, with a mean age of 6.15±2.05 years. No statistically significant differences were observed in terms of gender (p=0.914), age (p=0.375), or disease course (p=0.146) between the two groups, indicating comparability (Table-I).

**Table I:** Comparison of the general data between the two groups.

Group	Sample Size	Gender (Male/Female)	Age (years)	Course of Disease (d)
Study Group	103	56/47	6.40±1.93	3.25±1.08
Control Group	97	52/45	6.15±2.05	3.02±1.15
*t/χ^2^Value*		0.012	0.888	1.459
*P Value*		0.914	0.375	0.146

The levels of MP-DNA, IL-10, and IL-6 in the study group and control group were compared. The levels of MP-DNA (t=29.033; p<0.0001), IL-10 (t=18.233; p<0.0001), and IL-6 (t=28.692; p<0.0001) in the study group were significantly greater than those in the control group.Table-II

**Table II:** Comparison of MP-DNA, IL-10, and IL-6 levels between the study group and the control group (*x̄*±*s*).

Group	Number of Cases	MP-DNA (copy/mL)	IL-10 (pg/mL)	IL-6 (pg/mL)
Study Group	103	5.32±1.16	35.72±10.46	92.45±23.18
Control Group	97	1.74±0.37	14.28±5.12	21.36±7.85
*t Value*		29.033	18.233	28.692
*P Value*		0.000	0.000	0.000

Children with MPP were divided into good and poor prognosis groups on the basis of their outcomes at the three month follow-up. As shown in Table-III, among the 103 children in the study group, 82 had a good prognosis, and 21 had a poor prognosis. The levels of MP-DNA (t=27.174; p<0.0001), IL-10 (t=8.400; p<0.0001), and IL-6 (t=8.981; p<0.0001) in the poor prognosis subgroup were significantly greater than those in the good prognosis subgroup.

**Table III:** Comparison of MP-DNA, IL-10, and IL-6 Levels among patients with Different Prognoses in the Study Group (*x̄*±*s*).

Group	No. of Cases	MP-DNA (copy/mL)	IL-10 (pg/mL)	IL-6 (pg/mL)
Good Prognosis Group	82	2.02±0.75	30.15±8.23	75.32±19.64
Poor Prognosis Group	21	7.95±1.32	48.62±11.57	122.86±28.35
*t Value*		27.174	8.400	8.981
*P Value*		0.000	0.000	0.000

Further analysis of risk factors for poor prognosis of MPP was performed using poor prognosis as the dependent variable and indicators with statistically significant differences in Table II and Table-III as independent variables; the above indicators were included in a logistic analysis model. The results confirmed that high levels of MP-DNA (95%CI: 4.298~7.633; p<0.05), IL-10 (95%CI: 3.026~9.374; p<0.05), and IL-6 (95%CI: 2.582~6.044; p<0.05) were important risk factors for poor prognosis in MPP children (Table-IV).

**Table IV:** Analysis of Risk Factors for Poor Prognosis of MPP.

Variable	B Value	S.E.Value	Waldχ2Value	P Value	OR Value	95%CI
MP-DNA	1.745	0.484	13.003	<0.05	5.728	4.298~7.633
IL-10	1.673	0.570	8.611	<0.05	5.326	3.026~9.374
IL-6	1.374	0.395	12.097	<0.05	3.950	2.582~6.044
Pulmonary consolidation	0.070	0.194	0.130	>0.05	1.072	0.742~1.550
Duration of fever	0.428	0.256	2.796	>0.05	0.652	0.325~1.307
Pleural effusion	0.613	0.317	3.739	>0.05	0.542	0.250~1.174
Number of bronchoscopy treatments	0.126	0.244	0.265	>0.05	1.134	0.712~1.806
Length of hospital stay	0.105	0.165	0.404	>0.05	0.900	0.507~1.599

**Fig. 1:**
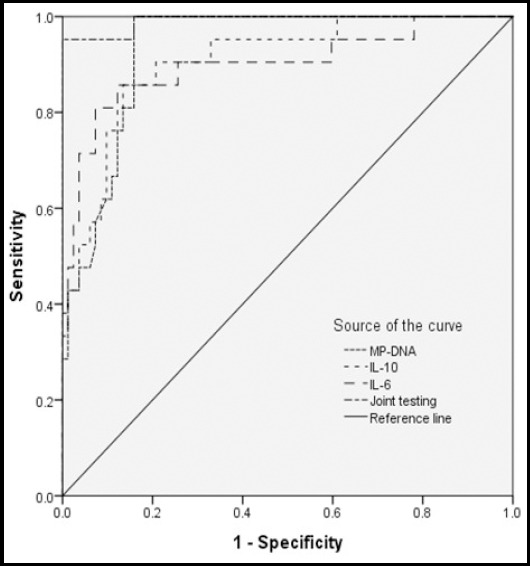
ROC Curve of the Predictive Model for Poor Prognosis in Mycoplasma pneumoniae Pneumonia (MPP).

On the basis of the logistic analysis results, the ROC curve results for MP-DNA, IL-10, and IL-6 are shown in Table V. The ROC curve of the poor prognosis prediction model for MPP patients was plotted using SPSS, as shown in Fig.1. The sensitivity, specificity and AUC of the combined detection of MP-DNA, IL-10, and IL-6 were 95.24%, 95.12%, and 0.992 (95%CI: 0.977–1.000; p<0.001), respectively, which were greater than those of MP-DNA alone (sensitivity 80.95%, specificity 85.37%, AUC 0.932), IL-10 alone (sensitivity 80.95%, specificity 86.37%, AUC 0.908), and IL-6 alone (sensitivity 85.71%, specificity 75.61%, AUC 0.900), as shown in Table-V.

**Table V:** ROC Curve Parameters.

Variable	AUC	SE	P	95% CI	Sensitivity	Specificity
MP-DNA	0.932	0.023	<0.001	0.886~0.978	80.95%	85.37%
IL-10	0.908	0.035	<0.001	0.839~0.977	80.95%	86.37%
IL-6	0.900	0.046	<0.001	0.810~0.990	85.71%	75.61%
Combined Detection	0.992	0.008	<0.001	0.977~1.000	95.24%	95.12%

## DISCUSSION

This study retrospectively analyzed the clinical data of two hundred children suspected of having *Mycoplasma pneumoniae* pneumonia (MPP); clarified the expression characteristics of MP-DNA, interleukin-6 (IL-6), and interleukin-10 (IL-10) in MPP children; and confirmed their important predictive value for the prognosis of MPP children. The results revealed that the levels of MP-DNA, IL-10, and IL-6 in the study group were significantly greater than those in the control group. The levels of the above indicators in the poor prognosis group were greater than those in the good prognosis group, and the high expression of all three genes was an independent risk factor for poor prognosis in MPP children. The area under the curve (AUC) of MP-DNA, IL-10, and IL-6 combined for predicting poor prognosis in MPP children reached 0.992, which was significantly greater than the efficacy of each indicator detected separately. These results elucidate the intrinsic correlation between relevant indicators and the onset and prognosis of MPP from the perspectives of pathogen infection and inflammation regulation, providing a key reference for clinical diagnosis and treatment.

In this study, the finding that serum MP-DNA levels are significantly increased in children with MPP is consistent with the results of many studies at home and abroad. Yan et al.^15^ reported that the incidence rate of MPP in children in China is increasing annually and that the MP-DNA load is closely related to the severity of infection, which is consistent with the conclusion that the high expression of MP-DNA in this study suggests that MP is actively replicated in vivo and that airway epithelial damage is aggravated. Wang et al.^16^ compared the value of multiplex PCR and serological detection in the diagnosis of MPP in children and confirmed that MP-DNA detection has higher sensitivity and specificity and can directly reflect the presence of pathogens, further verifying the rationality of using MP-DNA as a core diagnostic marker for MPP in this study.

In terms of cytokine expression characteristics, Biljana Medjo et al.^17^ reported that serum IL-10 levels were significantly elevated in children with MPP, while IL-4 levels did not significantly change, indicating that IL-10 plays a core regulatory role in the MPP immune inflammatory response, which is consistent with the results of abnormally high expression of IL-10 in this study. Tian et al.^18^ analyzed the differences in cytokines between children with MPP-induced lobar pneumonia and those with bronchopneumonia and confirmed that IL-6 levels were positively correlated with the degree of lung inflammation infiltration, which also supports the conclusion of this study that IL-6 is involved as a proinflammatory factor in the pathological damage of MPP.

However, this study revealed that children with MPP exhibit synchronously high expression of IL-6 and IL-10, which is different from the conclusion proposed by Ding et al.^19^ that “severe MPP children have insufficient IL-10 response”. This difference may be related to the different disease stratifications of the research subjects: Ding et al.’s study focused on children with severe MPP, whereas this study included children with MPP with different degrees of disease. Children with mild MPP have more active anti-inflammatory compensatory mechanisms involving IL-10, thus exhibiting high IL-10 expression. In addition, different detection time points may also lead to differences in results.

In this study, the cytokine levels of children at admission were measured, and Ding et al. focused on the dynamic changes in IL-10 levels during the course of the disease. These findings also suggest that cytokine detection needs to be comprehensively judged on the basis of the course of the disease. In terms of the prognostic value of indicators, the conclusions of this study are consistent with those of relevant research, and innovative breakthroughs have been made. Zhang et al.^9^ confirmed that the MP-DNA load is closely related to the risk of severe MPP in children and can be used as an indicator of disease severity. This finding is consistent with the conclusion in this study that high expression of MP-DNA is an independent risk factor for poor prognosis; Bao et al.^20^ analyzed the correlation between IL-6, procalcitonin (PCT), and the severity of MPP in children and reported that IL-6 levels were positively correlated with hospital stay, further supporting the prognostic value of IL-6 in this study.

The innovation of this study lies in the first combination of MP-DNA, IL-10, and IL-6 to construct a prognostic prediction model for children with MPP, which significantly improved the predictive efficiency. Compared with single-indicator testing, combined testing comprehensively evaluates the condition from three dimensions: pathogen infection load, proinflammatory response intensity, and anti-inflammatory regulatory ability. This approach can effectively avoid the limitations of single indicators, such as the imbalance between IL-6 and IL-10 in some children with low MP-DNA loads, indicating that their disease progression is driven by immune disorders. At this time, combined testing can effectively identify such high-risk children. This research fills the gap in the limitations of Fan et al.^21^ in predicting the prognosis of patients with MPP with a single inflammatory marker and provides a more accurate tool for clinical prognosis assessment.

### Strengths:

The advantages of this study are reflected mainly in three aspects: First, the research design is rigorous, using clinical comprehensive diagnosis and *Mycoplasma pneumoniae* culture as the gold standard for grouping, minimizing grouping bias to the greatest extent possible; second, the selection of indicators is comprehensive, including pathogen markers and immune inflammatory markers, achieving a dual analysis of “etiology pathology; and third, a joint prediction model was constructed, and its high predictive performance was verified through receiver operating characteristic (ROC) curve analysis, demonstrating strong clinical applicability. In addition, for the first time, this study revealed an association between high expression of IL-10 and poor prognosis in children with MPP. Previous studies have focused mainly on the anti-inflammatory effects of IL-10, but through logistic regression analysis, this study confirmed that excessively elevated IL-10 can inhibit the body’s ability to clear MP, leading to persistent infection. These conclusions help elucidate the mechanism of action of IL-10 in MPP and provide new targets for immune regulation therapy.

The new information added to the medical literature in this study includes two main points: First, elucidating the synergistic mechanism of MP-DNA, IL-10, and IL-6 in the pathogenesis and prognosis of MPP, confirming that immune inflammation imbalance is a key factor in disease progression; second, an efficient joint prediction model has been established, providing a reliable method for the early identification of high-risk children with poor prognosis in clinical practice. From a clinically relevant perspective, the results of this study have important practical value: at the diagnostic level, the combined detection of MP-DNA, IL-10, and IL-6 can improve the accuracy of MPP diagnosis, especially for atypical cases; at the level of disease assessment, by detecting the levels of the above indicators, the inflammatory status and severity of infection can be quickly determined; at the treatment level, targeted immune regulation therapy can be considered for children with IL-6 and IL-10 imbalance to improve prognosis.

### Limitations

There are several limitations to this study, and further research is needed in the following areas in the future. First, this is a single-center retrospective study with a relatively limited sample size and did not include mild pediatric patients in outpatient clinics, which limits the extrapolation of the results. Second, this study detected only the levels of indicators at the time of admission and did not conduct dynamic monitoring of the disease course, making it difficult to clarify the correlation between changes in indicators and treatment response. Finally, this study did not delve into the mutual regulatory mechanisms among the three. In summary, this study identified the expression characteristics and prognostic value of MP-DNA, IL-10, and IL-6 in children with MPP, confirming that their combined detection has high clinical application value. This research not only contributes to the study of the pathological mechanism of MPP but also provides a scientific basis for early clinical diagnosis, disease assessment, and personalized treatment, which is highly important for improving the diagnosis and treatment of MPP in children.

## CONCLUSIONS

Serum levels of MP-DNA, IL-10, and IL-6 are significantly elevated in children with MPP, and their high expression is an independent risk factor for poor prognosis in these children. The combined detection of MP-DNA, IL-10, and IL-6 has high predictive value for adverse prognosis in children with MPP, providing scientific references for early clinical assessment of disease severity, early warning of prognostic risks, and the formulation of individualized treatment plans.

### Recommendations:

In the future, multicenter, large-sample prospective studies can be conducted to include children with different disease severities and age groups to further verify the reliability of the conclusions. The value of dynamically monitoring MP-DNA, IL-10, and IL-6 levels can be explored to guide the adjustment of treatment plans. In vitro cell experiments can clarify the signaling pathway interaction between IL-6 and IL-10 after MP infection, providing a more solid theoretical basis for targeted therapy.

### Author’s contributions:

**YC:** Literature search, study design and manuscript writing.

**JW and ZC:** Data collection, data analysis and interpretation.

**YC:** Manuscript revision and validation and is responsible for the integrity of the study.

All the authors have read and approved the final manuscript.
